# Trade-offs between gene expression, growth and phenotypic diversity in microbial populations

**DOI:** 10.1016/j.copbio.2019.08.004

**Published:** 2020-04

**Authors:** Juhyun Kim, Alexander Darlington, Manuel Salvador, José Utrilla, José I Jiménez

**Affiliations:** 1 Faculty of Health and Medical Sciences, University of Surrey, Guildford, GU2 7XH, United Kingdom; 2 School of Engineering, University of Warwick, Coventry, CV4 7AL, United Kingdom; 3 Centre for Genomic Sciences, Universidad Nacional Autónoma de México, Campus Morelos, Av. Universidad s/n Col. Chamilpa 62210, Cuernavaca, Mexico

## Abstract

Bacterial cells have a limited number of resources that can be allocated for gene expression. The intracellular competition for these resources has an impact on the cell physiology. Bacteria have evolved mechanisms to optimize resource allocation in a variety of scenarios, showing a trade-off between the resources used to maximise growth (e.g. ribosome synthesis) and the rest of cellular functions. Limitations in gene expression also play a role in generating phenotypic diversity, which is advantageous in fluctuating environments, at the expenses of decreasing growth rates. Our current understanding of these trade-offs can be exploited for biotechnological applications benefiting from the selective manipulation of the allocation of resources.

## Highlights

• Limitations in molecular resources for gene expression influence bacterial physiology.
• Bacteria optimise trade-offs between resource allocation and growth.
• Resource allocation plays a role in the emergence of phenotypic heterogeneity.
• Trade-offs between bet-hedging and growth can be harnessed in biotechnology.

## Graphical abstract

**Figure:**
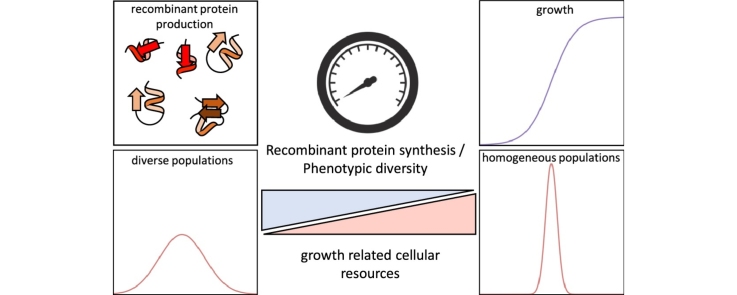

**Current Opinion in Biotechnology** 2020, **62**:29–37

This review comes from a themed issue on **Environmental biotechnology**

Edited by **David R Johnson** and **Stephan Noack**

For a complete overview see the Issue and the Editorial

Available online 1st October 2019

**https://doi.org/10.1016/j.copbio.2019.08.004**

0958-1669/© 2019 The Author(s). Published by Elsevier Ltd. This is an open access article under the CC BY license (http://creativecommons.org/licenses/by/4.0/).

## Introduction

The limited availability of some of the essential components of bacterial cells has a significant impact on the physiology of these organisms [1]. In an *Escherichia coli* cell, the number of RNA polymerases ranges from 1.5 to 11.4·10^3^ molecules, while there are between 6.8 and 72.0·10^3^ ribosomes (for growth rates in the range of 0.6–2.5 hour^−1^) [2]. These numbers depend on the environment but, even in those that are favourable to promote cell growth, there is always an upper limit given by the availability of one or more cellular components. This imposes a constraint on the functions that a cell can carry out at any given time. In fact, a cell can be understood as a closed economic system, in which strategies for an optimised allocation of resources are under positive selection in order to allow for survival under one – or more – environmental conditions.

The study of cellular components that can be limiting and the trade-offs emerging from these limitations has raised considerable interest in recent times [3]. This is due to their impact on the capacity of the cell for carrying out functions of biotechnological interest. Driven mainly by synthetic biologists, it has become clear that a better understanding of the rules governing the allocation of precious cellular resources is required in order to engineer complex cellular behaviours, such as the expression of recombinant biochemical pathways and circuits for artificial cellular computations [4].

In the case of bacteria, the allocation of resources may change from individual to individual leading to stochasticity in the different functions that a given cell can perform [5]. Cell-to-cell variation creates, even in the case of a clonal isogenic population, a phenotypic diversity at the population level. This may confer a selective advantage; especially when the environmental conditions fluctuate. Here we review our current understanding of how scarce molecular elements have an impact on gene expression at the single-cell level, which eventually generates the specialisation of different members of a population. We also explore how our current understanding of mechanisms contributing to phenotypic diversity can be harnessed for the improvement of biotechnological applications.

## Molecular factors limiting gene expression

**Figure 1:**
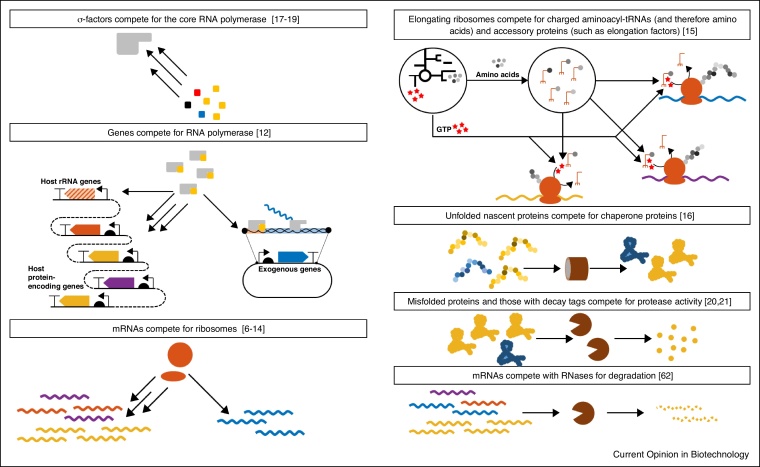
Molecular factors that can become limiting and affect gene expression. The cartoon summarises different components involved in the synthesis and degradation of mRNA and proteins that can become limiting depending on different environments (references describing supporting experimental evidence are shown in brackets).

Recent advances in both systems and synthetic biology have investigated the effects on gene expression which arise from limitations in the different parts of the machinery involved in the synthesis of mRNA and proteins, as well as in their degradation (Figure 1). During balanced exponential growth (i.e. when cells are growing maximally) the concentration of RNA polymerases and ribosomes is constant and this creates a set of fixed limited gene expression resources which must be distributed across all genes being co-expressed [6,7]. This results in competition between genes for their expression. As one gene is activated it sequesters resources from the pool of resources decreasing the expression of other genes as a consequence. Experimental evidence shows that, while both RNA polymerase and ribosomes represent limited gene expression resources, in most cases, it is the level of free ribosomes that imposes the greatest limitation on gene expression [6, 7, 8, 9, 10, 11, 12, 13, 14]. Additional evidence suggests that, in addition to ribosomal limitations, other translational resources, such as transfer RNAs, chaperone proteins and amino acid supply also form limited resources [15,16^•^].

While the transcriptional machinery becomes limiting when ribosomal sequestration is low [12], competition for the core RNA polymerase by σ-factors is key to modulating cellular stress responses. In *E. coli* most ‘housekeeping’ genes are expressed from promoters which respond to the σ^70^-core RNA polymerase complex while other σ-factors function as master regulators of key stress responses such as heat shock or entry into stationary phase. Competition between the σ^70^ factor and stress associated σ factors is proposed to be a key cause of the transition to these stressed states [17, 18, 19]. In addition to transcription and translation, the saturation of the cell's protease activity can impact the dynamics of gene expression especially for those proteins requiring high degradation rates [20,21]. Some authors have pointed to other limitations to cell physiology due to lack of space in the membrane for the respiratory machinery [22], as well as to the lack of cytosolic space and solvent capacity [23].

## The interplay between resource allocation and growth

**Figure 2:**
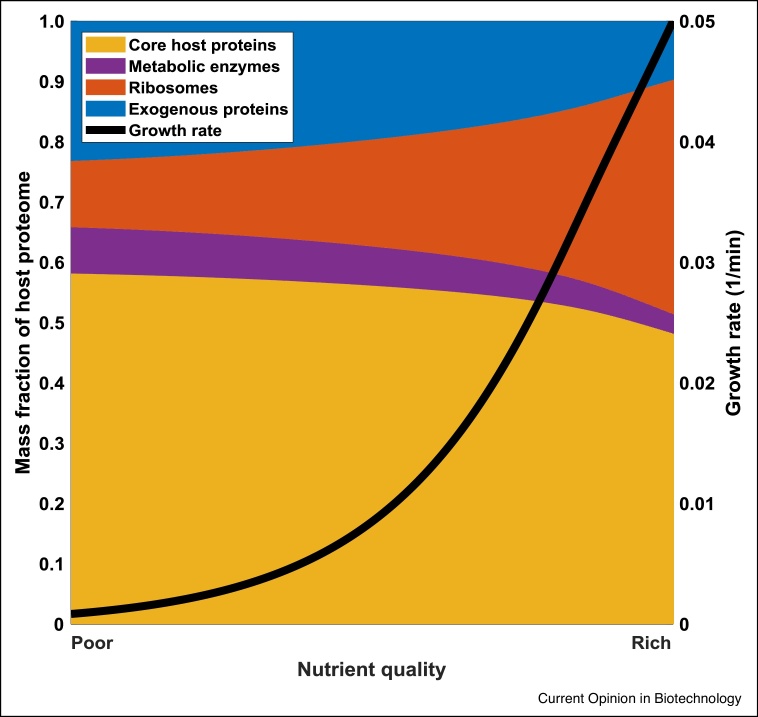
Partition of the bacterial proteome. The bacterial proteome consists of a largely growth-independent fraction containing the core host proteins required for cell maintenance. The remaining fraction is partitioned into translational resources (ribosomes and other associated proteins) and metabolic proteins such as transporters and enzymes. The core fraction is constant regardless of nutrient quality while the remainder of the proteome redistributes; when nutrient quality is high (rich medium), cells distribute more proteome to ribosomes (i.e. the ribosome-limited growth regime), whereas in poor medium (when cells are growing in a metabolism-limited growth regime) more of the fraction is allocated to metabolic proteins. The differential proteome partition results from non-linearities in ribosomal allocation [29, 30, 31]. Growth rate is determined by the distribution pattern of the proteome. Exogenous genes compete with this variable fraction for their expression. This dynamic partitioning is captured in the ordinary differential equation model described in [27]. The figure shows simulations of this model with nutrient quality n_s_ varied on a log scale between 0.01 and 5 representing poor and rich nutrients respectively. The pathway proteins where parameterised in the same way as host proteins but with a mRNA birth rate of 25 mRNAs per min. All other parameters are as determined in Ref. [27] but with *k_H_* set to 10^5^ molecules.

The cellular budget, typically defined as the amount of transcription and translation machinery, is not constant and varies with growth conditions [8]. This is the consequence of changes in the synthesis rate of the gene expression machinery, while the space available for transcription and translation remains unchanged owing to the constant ratio between the size of the nucleoid and the cell volume [24]. The interplay among growth, cellular resources and gene expression is non-linear and has been the subject of thorough study in recent years (Figure 2).

It has been demonstrated that the concentration of the RNA polymerase can tune the growth rate of *E. coli* [25]. This has been achieved through the conditional expression of the beta subunit (*rpoB*) placed under the control of an external inducer. Moreover, the growth rate seems to be hypersensitive to the RNA polymerase concentration [26]. The translational machinery also plays a key role in the interplay between resource allocation and growth. Fast growing cells produce more ribosomes as evidenced by a positive correlation between the ribosomal protein fraction and the growth rate [8,27]. However, this does not result in increased ribosome availability for the translation of all mRNAs due to the differential partitioning of the ribosomal pool into fractions allocated to produce different types of proteins. The size of these fractions depends on the growth rate, with faster growing cells allocating most of their ribosomes into the production of more ribosomal proteins [8,28] (Figure 2). Cellular biosynthesis capacity is primarily used for synthesising ribosome and ribosome affiliated proteins such as EF-Tu when nutrients are abundant, whereas it is mainly distributed to metabolic proteins in nutrient-poor medium to increase the supply of energy and amino acids [8]. The expression of synthetic genes decreases with increasing growth rate due to the prioritisation of ribosome production.

The above observations relate to carbon-limited cultures during exponential growth (when protein production is at steady state), but this does not seem to be representative of all possible strategies of optimisation of translation. For instance, recent work shows that the trade-offs in gene expression in *E. coli* may vary depending on whether the cells suffer limitations on carbon, nitrogen or phosphate [32^••^]. In phosphate-limited cultures the translational machinery is limited by the synthesis of ribosomes, while in nitrogen-limited conditions translation elongation rates become limiting due to lack of glutamine which leads to ribosomal stalling [32^••^].

The strategies for resource allocation may change over time even if growth conditions remain constant. For instance, it has been demonstrated that after a few (∼3) generations of producing an unneeded protein, *E. coli* cells optimise their investment in the production of the translational machinery using a mechanism that depends on the intracellular concentration of the alarmone (p)ppGpp [33,34]. In the presence of high concentrations of (p)ppGpp, most transcriptional resources were concentrated on nonribosomal promoters, whereas at low (p)ppGpp concentrations the allocation of the resources was directed to ribosomal promoters (and hence ribosome production). Ribosome production peaked during the early exponential phase along with low (p)ppGpp accumulation, which leads to maximum growth rate before ribosomal synthesis was tuned to cope with the load of additional protein production. A recent study also reported that the (p)ppGpp is crucial to maintaining the optimal growth rate [35^••^]. In this work, the concentration of (p)ppGpp was artificially controlled by conditionally expressing RelA responsible for the synthesis of the alarmone, or the recombinant hydrolase Mesh1 capable of (p)ppGpp degradation. The resulting strain exhibited a growth rate dependence on (p)ppGpp; however, allocation of resources was suboptimal compared to the wild-type [35^••^].

Considering the correlation between growth rate and number of ribosomes, it is generally assumed that species harbouring a large number of rRNA gene operons are endowed with faster growth rates [36]. This positive correlation has also been systematically investigated in *E. coli* under lab-controlled conditions. Gyorfy and colleagues constructed isogenic *E. coli* strains with different copy numbers of the *rrn* operon (from 5 to 10) and performed growth competition assays with the strains compared to the wild type [37]. Interestingly, they found that strains with lower numbers of *rrn* operons (5 and 6) outcompeted the wild-type as well as other isogenic strains in stable culture with minimal medium. In contrast, seven or eight copies of the *rrn* operon in a cell provided optimal growth in fluctuating conditions in a *rich* medium. The authors point to the burden of producing extra rRNAs as the potential reason for the disadvantage in nutrient-limited conditions whereas a surplus of rRNAs could enable better adaptations in the presence of nutritional upshifts. These results show that genetic information alone might not be enough to capture the complexity of the trade-offs between resources and growth resulting from the multilevel selection of microorganisms in the environment. In fact, experiments with native soil bacteria show that features typically associated with fast-growing organisms, such as the number of *rrn* operons and a reduced genome size are unrelated to growth rates in their natural environments in which nutrients are scarce. However, when soils are supplemented with additional nutrients, the expected positive correlations between *rrn* operons (or reduced genomes) and growth rates are observed [38^••^].

## The effects of molecular competition

The low availability of the molecular components involved in the synthesis of mRNA, proteins and their decay, is one of the factors contributing to the stochasticity leading to cell-to-cell phenotypic variations in microbial populations [39,40]. Stochasticity in gene expression affects the microbial physiology of clonal populations in multiple ways and contributes towards and increased diversity in growth strategies including biofilm formation [41,42] and even aging [43^•^]. Natural heterogeneity has also been harnessed to improve bioprocesses in the absence of genetic modifications [44]. Cell-level physiological effects may stem from the noisy expression of a small subset of genes. For instance, fluctuations in the lactose operon lead to a diversity in growth rates of clonal populations. As described in the previous section, changing the growth rate has an impact on gene expression through the differences in resource levels [45].

Perhaps the best-known example of phenotypic diversity is the emergence of persister cells in microbial populations. These cells have low or negligible growth rates and are better suited to resist antibiotic treatments compared to their fast-growing counterparts [46]. Persisters are often linked to variation in the expression levels of toxin-antitoxin systems, such as the *mazEF* system of *E. coli*, which depend on resource allocation for their expression. The *mazEF* genes are capable of conducting their own post-transcriptional regulation that can increase the phenotypic heterogeneity in a population [47^•^].

Fluctuations in the expression of multiple genes in bacterial cells are responsible for a phenomenon called bet-hedging. Bacteria may allocate part of their gene expression machinery to the production of proteins that are not required under certain specific growth conditions [48]. Comparisons between quantitative proteomics data and predictions of a genome scale model of Metabolism and gene Expression (ME-model) have determined that up to 50% of the *E. coli* proteome is unused under a given condition [49]. This spurious activation takes place at the expense of decreasing the growth rate but is particularly useful in preparation for environmental changes [50^••^]. A pioneering work demonstrated that bet-hedging strategies facilitate the adaptation to different carbon sources in cultures of *Lactococcus lactis* [51]. Likewise, Kotte *et al.* showed that a population of *E. coli* cells splits into two stochastically generated phenotypic subpopulations when exposed to a glucose–gluconeogenic substrate shift. This mechanism manifests as a fitness trade-off; only cells that refrain from growing very fast on glucose have the ability to switch to a gluconeogenic growth [52]. Bet-hedging is therefore often linked to the survival under conditions in which the specialisation of parts of the population can benefit the whole group. This is the case of *Paracoccus denitrificans*, which is a facultative anaerobe capable of using nitrate as an electron acceptor. In order to obtain energy, the cells need to produce four reductases that catalyse the reduction of nitrate to molecular nitrogen via nitrite, nitric and nitrous oxides. While all cells in a clonal population of *P. denitrificans* reduce nitrate, a bet-hedging strategy is used to produce a subpopulation of nitrite reducers that is preserved even when oxygen becomes available in preparation to future anoxic conditions [53^•^].

## Engineering resource allocation for biotechnological applications

**Figure 3:**
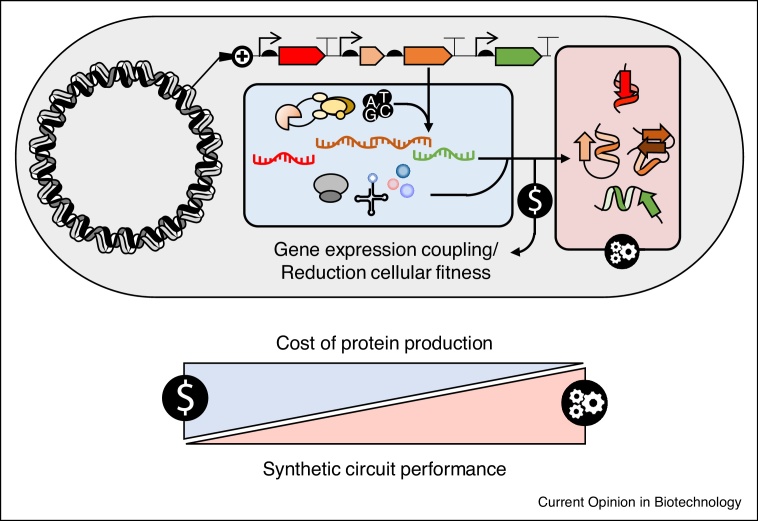
The impact of resource allocation on the performance of exogenous genetic constructs. The amount of resources allocated for the expression of recombinant genes is the result of the trade-offs between overall resource availability and growth. During fast growth the budget available for functions not related to growth is decreased, therefore making the synthesis of other proteins, such as those corresponding to exogenous functions, more expensive. This in turn negatively affects the performance of the synthetic circuits and pathways.

Building on the current understanding of how scarce resources influence strategies for gene expression, a number of studies have quantitatively investigated their impact on the expression of recombinant gene circuits. These works have unveiled interactions in seemingly unconnected genes: resources spent in one gene are not available for other genes producing undesired couplings in their expression [11,12,54, 55, 56] (Figure 3). Increasing awareness of host and environmental dependencies has led to systematic testing of simple circuits in a variety of host backgrounds (e.g. [57, 58, 59, 60]). It has been shown that circuit gene expression can vary up to 1000-fold depending on the host selected [58]. Together these studies do not identify a trend between circuit behaviour, strain genotype and/or growth conditions suggesting host-circuit interactions are highly dependent upon the specific conditions. A key cause of the strain and environmental context dependency is that both host genotype and growth conditions lead to differences in resource levels in bacteria.

Novel approaches have been developed to mitigate the burdensome effects that the exogenous genes impose in the host used for their expression and improve the predictability of gene expression [61]. Examples include the engineered *E. coli* strain with tunable transcriptional activity obtained through the conditional expression of *rpoB* [26], and the selective allocation of the mRNA degradation machinery [62]. Other studies have explored the use of engineered transcriptional and translational machinery by using feedback controllers that allow cells to operate in two regimes, a basal one in which the exogenous genes are not expressed, and another in which the mechanisms required to alleviate the burden associated to recombinant expression is required [3]. To this end it has been shown that is possible to repurpose the stress response mediated by σ^32^ in order to decrease the expression of the exogenous genes when the burden is above a certain level [16^•^]. Other works show that orthogonal molecular elements that can be selectively allocated for the transcription [63] or translation [64^•^] of genes of interest and can effectively reduce the burden in the host used for expression when used in conjunction with feedback controllers.

These examples have in common that they mitigate the negative impact of the exogenous genes by reducing their total protein output, which is a feature that may be incompatible with many biotechnological applications. An alternative approach is to maximise the exogenous protein output by decreasing the size of the non-essential proteome produced by the host, which is the basis of genome minimisation methods [65]. By removing parts of a genome that are not required under a specific growth condition, it is possible to free resources creating strains capable of both faster growth and greater recombinant protein production. For instance, the deletion of non-essential genes including prophages, some mobile genetic elements and flagella genes in *Pseudomonas putida* results in increased heterologous genes expression and higher intracellular concentrations of ATP and NADP(H) [66,67], as well as a higher bioplastic production [68] while maintaining cellular physiology in a defined medium.

**Figure 4:**
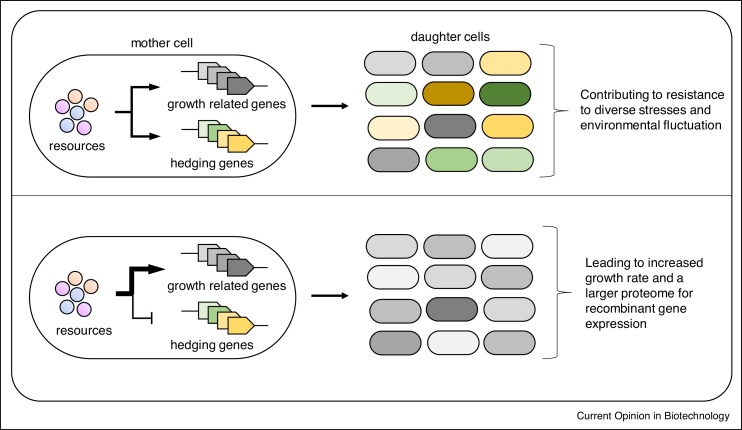
Selective manipulation of bet-hedging strategies. The removal of non-essential functions releases cellular resources that can be used to increase growth and protein production yields at the expense of reducing the phenotypic diversity in a clonal population.

Approaches such as genome minimisation and adaptive laboratory evolution (ALE) tap directly into the manipulation of bet-hedging strategies: improved gene expression can be obtained at the expenses of reducing the phenotypic diversity in a population (Figure 4). Releasing resources has the immediate effect of increasing recombinant gene expression at the expense of reducing the ability of a population to adapt to fluctuating environments. This has been addressed in a study in which *E. coli* were cultured continuously in a constant environment. This selected for strains carrying mutations in the RNA polymerase subunit *rpoB*, resulting in a considerable increase in growth rate in that environment. These mutants re-wired the transcriptional regulatory network and show a specialised proteome [69], with reduced overall stress anticipation functions and increased growth functions. The resulting strains showed less antibiotic persistence and a longer lag phase during a diauxic shift, consistent with the trade-off between bet-hedging and adaptation to a specific niche. Another example combines both approaches: *E. coli* MS56 obtained from MG1655 after the deletion of 1.1 Mbps was subject to ALE [70^•^]. While the minimised strain exhibited diminished growth, after 807 generations of continuous culturing in a constant environment, compensatory mutations emerged that resulted in a strain with a growth phenotype comparable to that of MG1655. The evolved strain displayed rearrangements in the metabolic, transcriptional and translational profiles, notably exhibiting a decreased ‘translational buffering’ (bet-hedging) compared to the wild-type strain.

Phenotypic diversity, however, may be advantageous in applications in which the protein products are toxic for the cell. Tan *et al.*, produced an *E. coli* strain that expresses the moderately toxic T7 RNA polymerase using a positive feedback loop [71]. Cells with high expression levels exhibited poor growth rates, which lead to protein accumulation due to lack of dilution, while cells with low expression levels grew faster. The result was a bi-stable population with high and low expressing cells. This and the examples discussed above show how phenotypic diversity can be factored in for different strategies depending on the final goal.

## Conclusions

The interplay between the allocation of resources, the expression of exogenous genes and growth is increasingly being considered when designing circuits, pathways and hosts for recombinant expression. This understanding is key to increase the complexity of the genetic circuits expressed in bacterial hosts and to leverage contemporary methods for building regulatory logics. There are, however, some limitations to our current capabilities to predict the behaviour of microorganisms subject to molecular competition. For instance, our understanding of resource allocation strategies in conditions other than carbon-limited balanced growth is small.

Although the research efforts to understand resource allocation in bacteria have been largely driven by the aim of maximising protein production, these investigations have also produced a wealth of knowledge about the fundamental principles underlying the multilevel selection of resource investment strategies. The trade-offs unravelled in the recent years point to mechanisms likely evolved in scarcity, in which phenotypic diversity emerging by low numbers of molecular components among other factors, plays an important role in the survival of microbial populations. Microbial cells are, however, also hardwired to maximise their fitness, and abundance of nutrients representative of lab conditions leads to the adaptation of microorganisms that are selected due to higher growth rates obtained through the loss of functions involved in maintaining population diversity. These properties could be harnessed to promote or hinder phenotypic diversity in a population for different applications.

## Conflict of interest statement

Nothing declared.

## References and recommended reading

Papers of particular interest, published within the period of review, have been highlighted as:

• of special interest
•• of outstanding interest
